# Vegetarianism and Cancer

**Published:** 1888-11-24

**Authors:** 


### VEGETARIANISM AND CANCER.

It was stated some time ago, on the authority of two distinguished French medical men, that "cancer is all but unknown among persons whose food is exclusively vegetable;" and that "the increase of cancer is largely due to the carnivorous habits of the present generation." It is very unfortunate that among scientifically-trained men there should be so many who are ready to jump to conclusions on insufficient evidence. One swallow does not make a summer, and one fact does not usually warrant a sweeping generalisation. Many doctors are twitted with excessive conservatism, and charged with being behind the times, because they do not fly into ecstasies over every new thing that is vaunted and puffed up. This statement about vegetarianism and cancer well illustrates the necessity for caution, and for the accumulation of a large number of similar facts before making broad and sweeping statements. It now turns out that not onlyis there no proof that vegetarianism is a preventive of cancer, but that in some cases it would be possible to make it appear that it was even a promoter of that terrible disease. Here, for example, is a set of facts which seem to tell quite an opposite story. In the Jeypore Hospital, from 1880 to 1887, operations for cancer were performed in 102 cases. All these were what are called "major" operations, that is, operations for serious disease in important parts of the body. Of the 102 persons thus operated upon, 41 were meat eaters, but 61 were strict vegetarians, and had been so from birth, on account of their caste. But that does not exhaust the significance of the facts, for these people were born of parents who belonged to non-flesh-eating castes. It is possible that the 61 vegetarians were descendants of many generations of purely vegetable feeders. If, therefore, a vegetarian diet had had anything to do with exemption from cancer, all the 61, with such a personal history and such inherited freedom from transmitted taint, should have been absolutely and entirely proof against any attack of the disease whatever. There is no need to push the other indication, that since only 41 of the 102 cancerous were among flesh-eating people, whilst 61 were among pure and hereditary vegetarians, therefore vegetarianism may be a cause of cancer. The circumstance was probably a mere coincidence. But the facts are of sufficient importance to make it desirable to follow up much more extensively than heretofore the particular line of observation thus pointed out.

